# Nerve entrapment syndromes of the upper limb: a pictorial review

**DOI:** 10.1186/s13244-022-01305-5

**Published:** 2022-10-12

**Authors:** Mohammad Danish Mangi, Steven Zadow, WanYin Lim

**Affiliations:** 1 Faculty of Health and Medical Sciences, The University of Adelaide, 4 North Terrace, Adelaide, SA 5000 Australia; 2 Dr Jones & Partners Medical Imaging, Prospect, SA 5082 Australia; 3 Department of Medical Imaging, Flinders Medical Centre, Flinders Drive, Bedford Park, SA 5042 Australia; 4 Department of Radiology, Royal Adelaide Hospital, Port Rd, Adelaide, SA 5000 Australia

**Keywords:** MRI, Ultrasound, Entrapment, Nerve

## Abstract

Peripheral nerves of the upper limb may become entrapped at various points during their anatomical course. While physical examination and nerve conduction studies are the mainstay of diagnosis, there are multiple imaging options, specifically ultrasound and magnetic resonance imaging (MRI), which offer important information about the potential cause and location of nerve entrapment that can help guide management. This article overviews the anatomical course of various upper limb nerves, including the long thoracic, spinal accessory, axillary, suprascapular, radial, median, ulnar, and musculocutaneous nerves, and describes the common locations and causes of entrapments for each of the nerves. Common ultrasound and MRI findings of nerve entrapments, direct or indirect, are described, and various examples of the more commonly observed cases of upper limb nerve entrapments are provided.

**Key points**

1. Ultrasound and MRI can illustrate the cause and location of nerve entrapments.
2. Ultrasound findings of nerve entrapment include proximal thickening and hypoechoic appearance.
3. MRI not only assesses the nerves but can also reveal the secondary effects of neuropathy such as downstream muscle denervation oedema or atrophy.

## Background

Peripheral nerves of the upper limb can become entrapped at various points [1]. While physical examination and nerve conduction studies are the mainstay of diagnosis, there are multiple imaging options, especially ultrasound and magnetic resonance imaging (MRI), which offer information about the cause and location of nerve entrapment, and help guide treatment [1].

Ultrasound is an appropriate first-line imaging modality for entrapment syndromes as it is cheap, fast, has high spatial resolution, and allows dynamic nerve assessment. The presence of an ultrasonographic Tinel’s sign, which refers to paraesthesia in the cutaneous distribution of the nerve in response to transducer pressure at the entrapment site, can be a helpful supportive finding [2]. Ultrasound also provides an opportunity for the operator to obtain more clinical information or history which may otherwise not be readily available. The drawbacks of ultrasound are that its field of view can be limited in depth and obstructed by structures such as bone. In addition, the technique is inherently operator dependent [2].

MRI of peripheral nerves predominantly utilises T1-weighted and fluid-sensitive sequences [2]. Magnetic resonance neurography (MRN) is a technique which utilises the longer T2 isolation time of nerves to better image them [3]. Benefits of MRI include better visualisation of deep nerves and high contrast resolution. Gadolinium-based contrast can also be administered to highlight mass lesions and inflammation that may be contributing to symptoms. The drawbacks of MRI are its cost, time-consumption, contraindications (e.g. pacemakers), and signal contamination from foreign bodies or blood flow [2].

This article describes the characteristic ultrasound and MRI findings of nerve entrapment syndromes, reviews the anatomy of the peripheral nerves of the upper limb, and illustrates common sites of entrapment. It is important for radiologists to be familiar with these entrapment syndromes as correctly diagnosing the site and cause of entrapment allows targeted treatments, such as corticosteroid injections or surgery, to be performed.

## Main text

On ultrasound, nerves are more echogenic than muscles and less echogenic than tendons [2, 4]. Along a nerve’s short axis, hyperechoic connective tissue, called epineurium, surrounds the hypoechoic nerve fascicles, giving a honeycomb appearance [4]. Along the long axis, nerves have a hypoechoic and coarse appearance [2, 4]. Nerves often travel alongside vessels and, hence, vessel identification can be a handy tool for locating a target nerve. With entrapment neuropathy, the nerve can be flattened at the site of pathology and proximally enlarged, thickened and hypoechoic. The normal honeycomb pattern of fascicles may appear disrupted as a result of intraneural oedema. Colour Doppler may reveal increased vascularity [2]. Potential causes of entrapment should be sought, for example mass lesions, tenosynovitis, foreign bodies, fibrous bands, and anatomical variants such as anomalous muscles [2]. Neuropathic muscular changes may not be evident acutely; increased muscle echogenicity may be seen within the first two weeks, with atrophy over the longer term [4].

On MRI, nerves are normally hypointense on T1-weighted images and isointense to hyperintense on fluid-sensitive sequences [1]. In entrapment neuropathy, MRI can reveal proximal enlargement and swelling with increased T2 signal [1]. There is flattening of the nerve at the site of entrapment [3]. Muscular changes are more easily appreciated on MRI than ultrasound, with denervation oedema acutely and atrophy in more longstanding cases [2]. Increased T2 signal alone may indicate the magic angle artefact, which is avoided by positioning the extremity at less than 30° relative to the main magnetic field [5].

### Overview of upper limb nerves and their entrapment syndromes

**Fig. 1:**
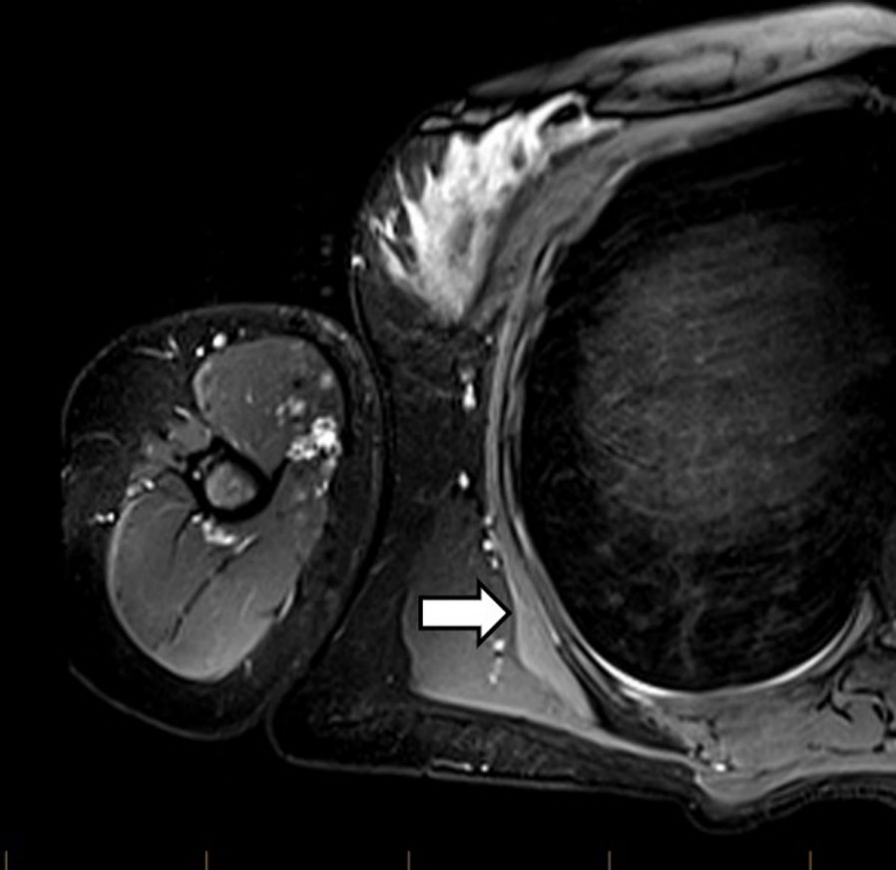
Axial proton density fat saturation (PDFS) image of a 30-year-old female who presented with medial scapular winging. There is denervation oedema of the serratus anterior (arrow) which is in the distribution of the long thoracic nerve (not shown)

The **﻿long thoracic nerve** arises from C5-C7 and runs anteriorly to the posterior scalene muscle. Its C5 and C6 roots run through the middle scalene muscle, whereas the C7 portion travels between the anterior and middle scalene muscles. It then courses laterally deep to the clavicle and superficial to the first two ribs and enters a fascial sheath, continuing along the midaxillary line to lie on the superficial surface of the serratus anterior muscle, which it innervates [6]. Neuropathy of the long thoracic nerve leads to medial winging of the scapula as a result of serratus anterior weakness and unopposed rhomboid action [6]. Potential sites of entrapment include in the middle scalene muscle and between the middle and posterior scalene muscles. More distally, compression can occur between the second rib and structures such as the clavicle, coracoid process, or the undersurface of the scapula. Traction of its fascial sheath can compress the nerve, particularly on raising the arm. A chest tube in the midaxillary line can be an iatrogenic cause of entrapment [6, 7]. Denervation oedema and atrophy of the serratus anterior may be noted on imaging (Fig. 1).

**Fig. 2:**
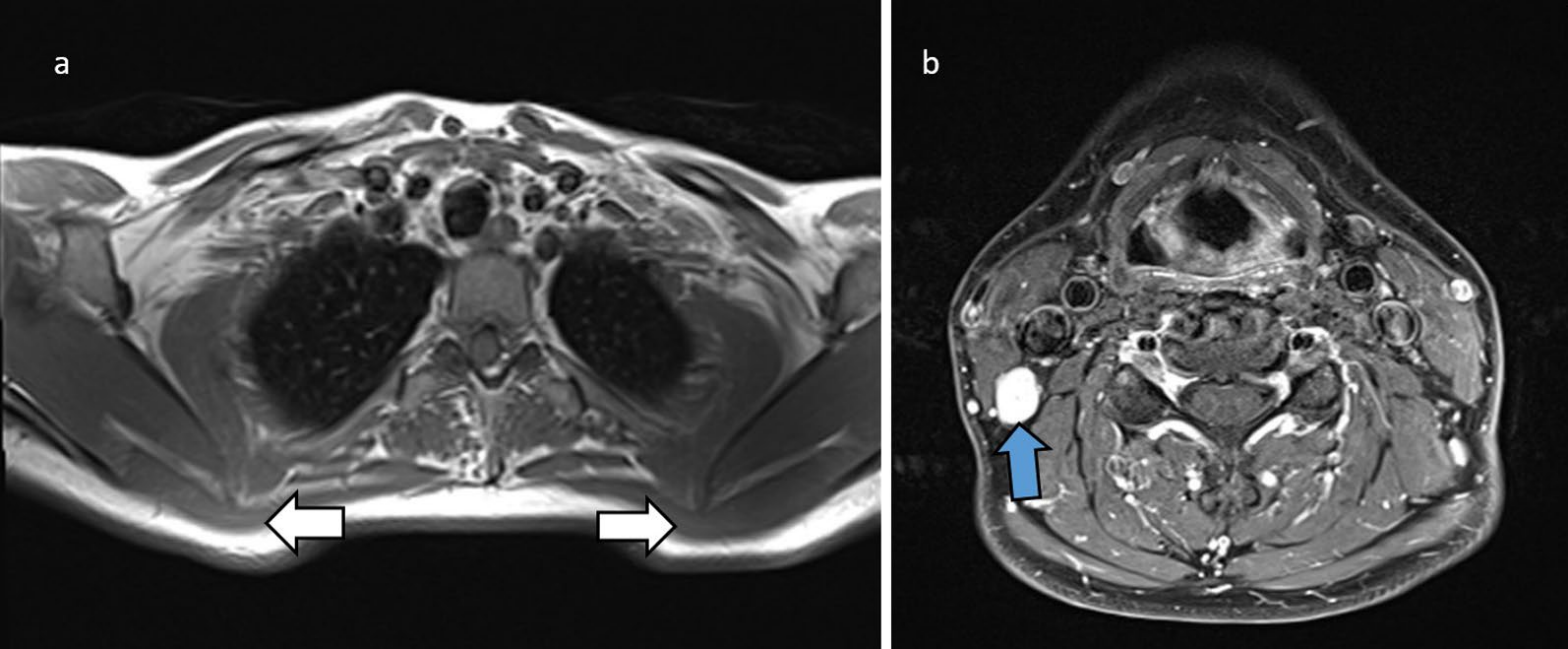
37-year-old female with lateral scapular winging after schwannoma excision. Axial T1 at the level of the thoracic inlet (**a**) demonstrates asymmetry of the trapezius muscles (white arrows) with right-sided volume loss. Axial T1 fat-saturated post-contrast study (**b**) shows the avidly-enhancing schwannoma, which was located at level 2B in the upper posterior neck, deep to the upper sternocleidomastoid muscle and immediately posterior to the right internal jugular vein (blue arrow). This is along the expected path of the accessory nerve. While the nerve lesion in this case was caused by surgery, the illustrated muscle changes are also seen in entrapment

The **spinal accessory nerve** arises from C1 to C6 and runs superiorly through the foramen magnum and across the posterior cranial fossa, before exiting the skull through the jugular foramen. It then descends near the jugular vein towards the sternocleidomastoid which it innervates, and then enters the posterior triangle of the neck, supplying the trapezius [8]. Entrapment produces scapular winging which is classically lateral compared to that of long thoracic nerve palsy. Entrapment can occur at the jugular foramen, for example due to tumour, or in the posterior triangle during interventions such as lymph node biopsies [8, 9]. Denervation oedema and atrophy of the sternocleidomastoid and trapezius should be sought on imaging (Fig. 2).

**Fig. 3:**
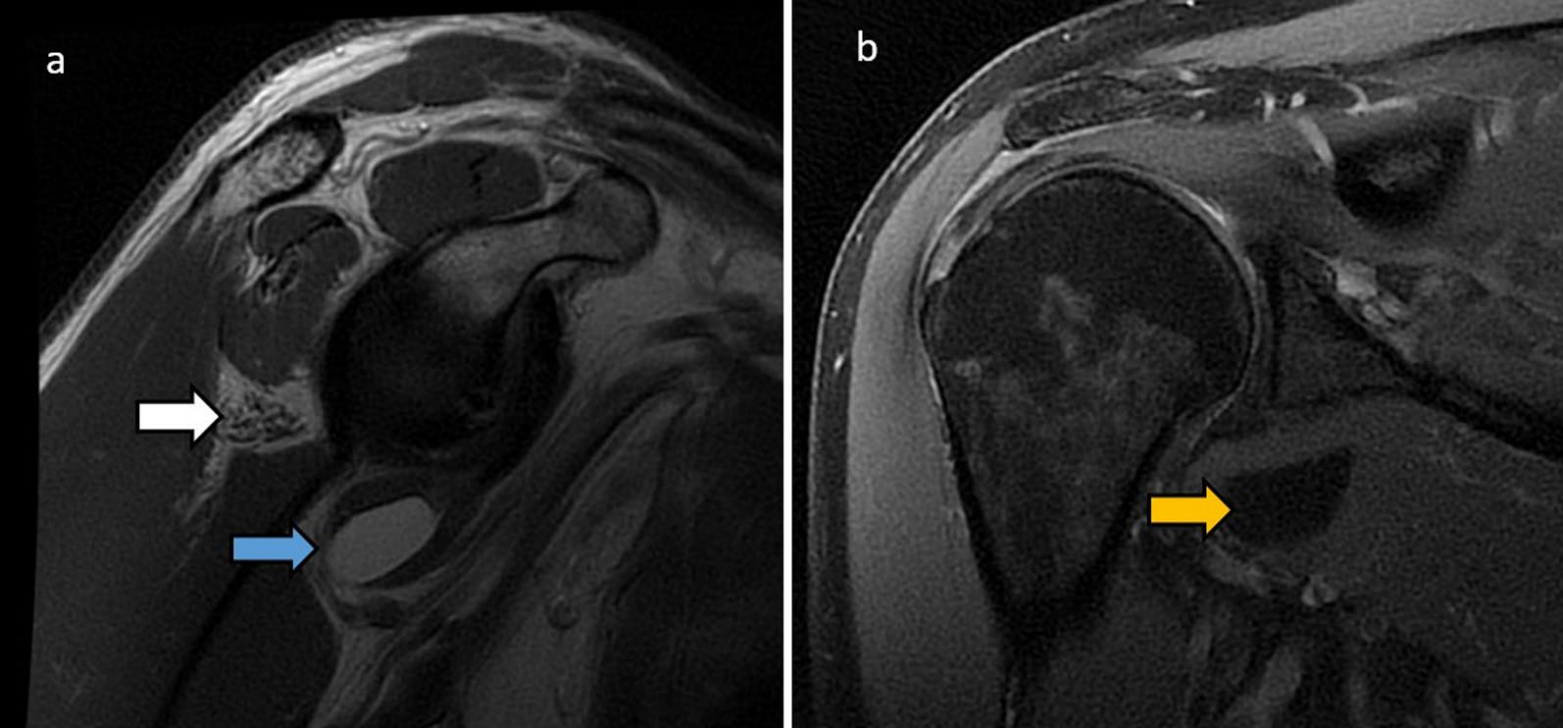
70-year-old with underwent MRI for rotator cuff tendinopathy. Sagittal proton density (PD) shoulder (**a**) shows severe fatty atrophy of the teres minor (white arrow). There is a lipomatous tumour with complete fat-suppression on the coronal PDFS sequence (**b**, yellow arrow), which is within the subscapularis muscle belly inferiorly and is impinging on the quadrilateral space and the traversing axillary nerve (**a**, blue arrow). This is consistent with chronic quadrilateral space syndrome

The **axillary nerve** arises from the posterior cord of the brachial plexus, with roots C5 and C6. It exits the axilla via the quadrilateral space which is bounded by the teres minor superiorly, teres major inferiorly, long head of triceps medially and proximal humerus laterally, and also contains the posterior humeral circumflex artery and vein. The axillary nerve then gives off three branches. The anterior branch supplies the anterior portion of the deltoid. The posterior branch supplies the teres minor and posterior deltoid before continuing as the upper lateral cutaneous nerve of the arm, which innervates the skin over the lateral deltoid. The articular branch supplies the anteroinferior glenohumeral joint [10]. At the shoulder, the axillary nerve can be compressed by tumours, large osteophytes of glenohumeral osteoarthritis, posteroinferior paralabral cysts, scapular fracture or anterior shoulder dislocation. Surgical procedures of the deltoid muscle may also injure the nerve [11]. The most common site of axillary nerve entrapment is the quadrilateral space (as shown in Fig. 3), where it can be compressed along with the posterior humeral circumflex artery. This quadrilateral space syndrome presents with paraesthesia over the cutaneous distribution of the axillary nerve, posterior shoulder pain, weakness of the deltoid and teres minor, and cyanosis and pallor of the distal limb [10]. Point tenderness over the quadrilateral space can be sought during ultrasound [11].

**Fig. 4:**
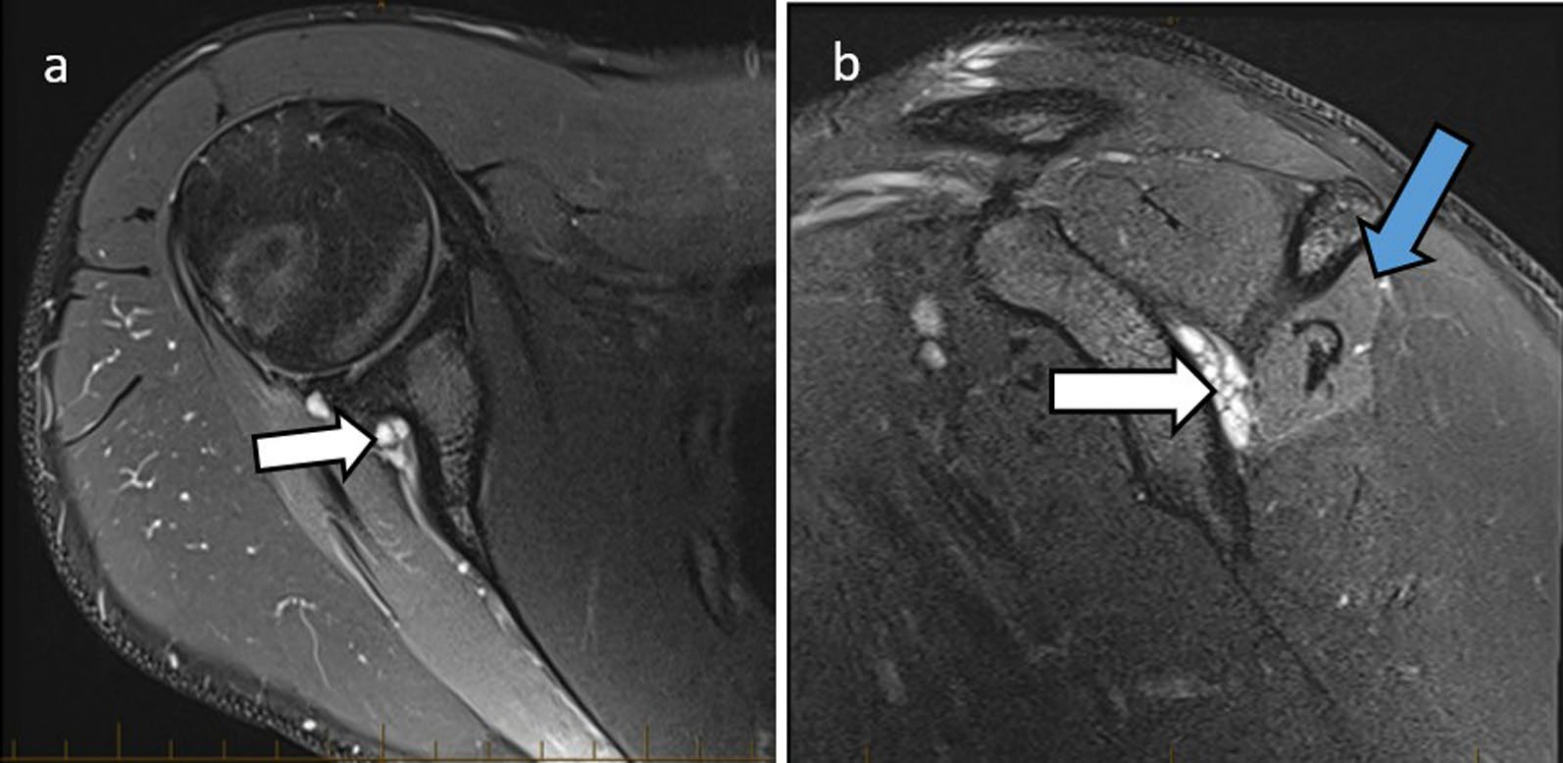
20-year-old male with 6 months’ history of shoulder weakness. Axial (**a**) and sagittal (**b**) PDFS sequences show a posterior labral tear with paralabral cyst (white arrows) extending into the spinoglenoid notch. There is atrophy and mild denervation oedema in the infraspinatus muscle (**b**, blue arrow), sparing the supraspinatus since the site of entrapment is distal to the supraspinatus innervation

**Fig. 5:**
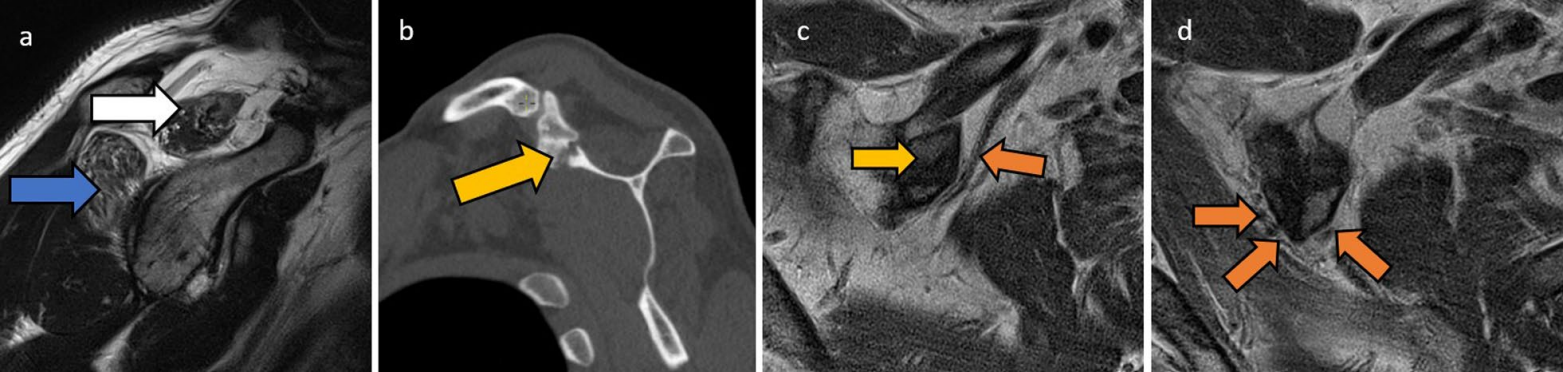
49-year-old male with right upper limb weakness. Sagittal PD (**a**) shows moderate-severe fatty atrophy of the supraspinatus (white arrow) and infraspinatus muscles (blue arrow) which is compatible with suprascapular nerve entrapment. Interestingly, the site of the impingement is not in the typical suprascapular groove, but more proximally, secondary to ossification along the coracoclavicular ligament which is seen on sagittal CT (**b**, yellow arrow). The patient had a history of acromioclavicular sprain. Consecutive axial PD images (**c**, **d**) show that the ossification (**c**, yellow arrow) contacted and displaced the suprascapular vessels and nerve (**c**, **d**, orange arrows) posteromedially, stretching the nerve

The **suprascapular nerve** arises in the upper trunk of the brachial plexus, with roots C5 and C6. It passes through the posterior triangle of the neck and traverses the suprascapular notch to enter the supraspinous fossa where it innervates the supraspinatus muscle. It then continues through the spinoglenoid notch to the infraspinous fossa, terminating in the infraspinatus muscle which it supplies. It also provides sensory supply to the glenohumeral and acromioclavicular joints [12]. The suprascapular notch and spinoglenoid notch are common sites of entrapment. At the suprascapular notch, anatomical variation of the subscapularis muscle fibres and anterior coracoscapular ligament and calcification of the suprascapular ligament and fractures of the suprascapular notch have been implicated in entrapment. At the spinoglenoid notch, compression may occur by enlargement of the inferior transverse scapular ligament and spinoglenoid notch veins. Suprascapular nerve entrapment presents with posterior shoulder pain and weakness of abduction and external rotation [12]. Figures 4 and 5 illustrate cases of suprascapular neuropathy.

**Fig. 6:**
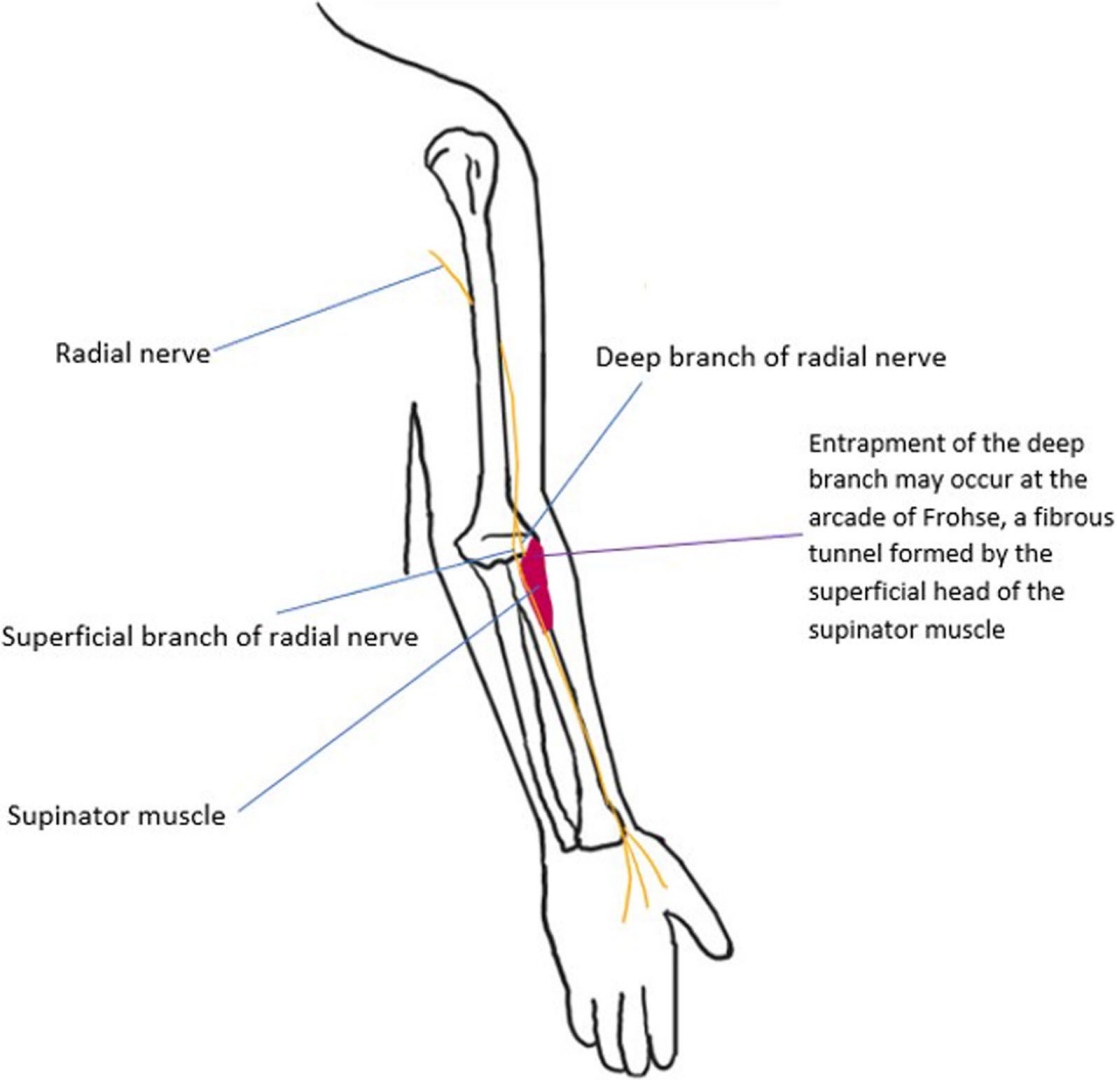
The radial nerve is shown. The most common entrapment neuropathy of the radial nerve is of the deep branch as it traverses the arcade of Frohse

**Fig. 7:**
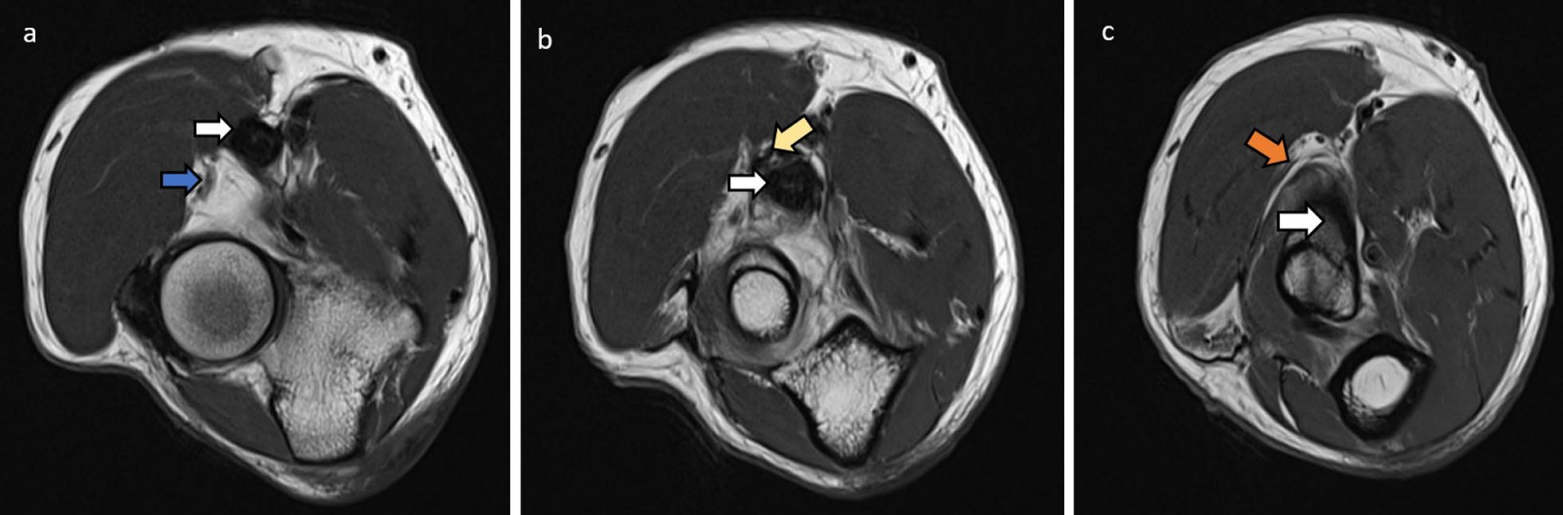
Stack of PD axials of the upper forearm of a 55-year-old male post distal biceps tendon repair. The biceps tendon (**a**, **b**, **c**, white arrows) is intact although thickened just proximal to the tunnel entry. The radial nerve above the elbow appears normal (**a**, blue arrow). The superficial branch (**b**, yellow arrow) comes close to scarring around the enlarged biceps tendon and appears adherent. The deep branch (**c**, orange arrow) continues without abnormality to the supinator

**Fig. 8:**
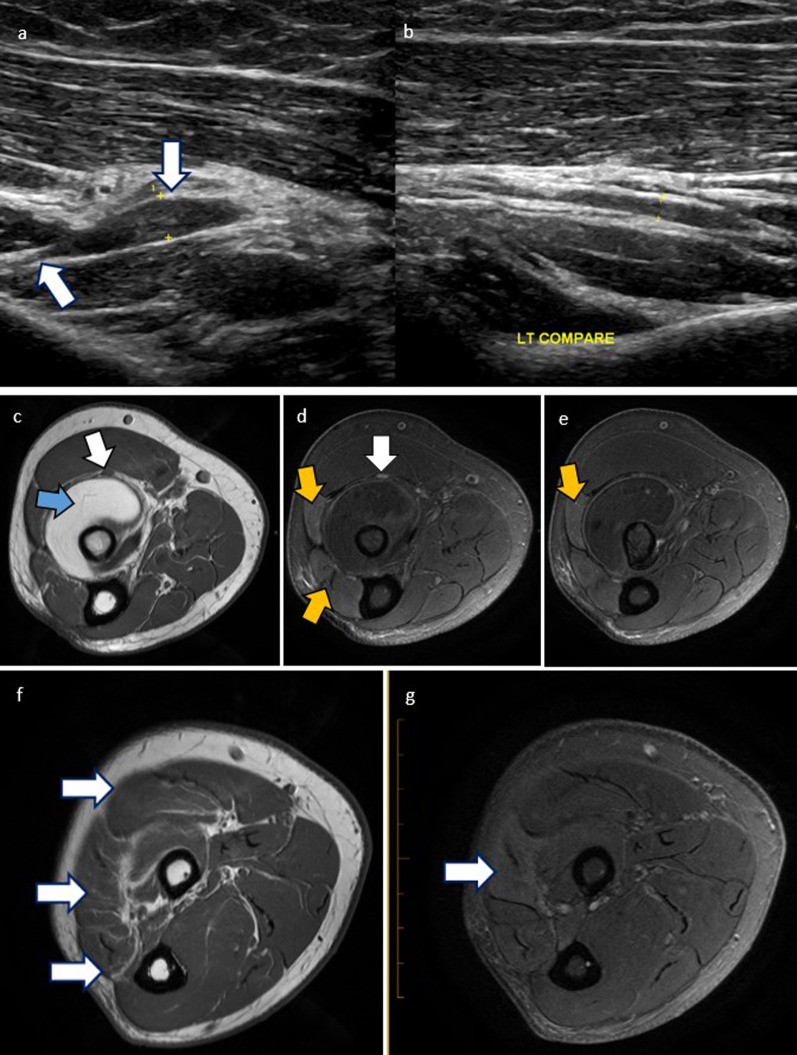
Cases of posterior interosseous nerve (PIN) pathology are illustrated: **a**, **b** Forearm ultrasound of a 58-year-old female who presented with arm pain. There is focal thickening throughout the right PIN (**a**, white arrows). This extends into the arcade of Frohse. This appearance can be seen with PIN syndrome. No adjacent collection or mass is identified. There was focal tenderness with transducer pressure. The left PIN is provided for comparison (**b**). **c**–**e** 60-year-old male who presented with progressive weakness of finger extension. Intramuscular lipoma of the supinator (blue arrow) resulted in PIN impingement at the arcade of Frohse (**c**). Axial T1 (**c**) and Axial PDFS images at the level of narrowing (**d**) and more distally (**e**), show focal thickening and hyperintensity of the PIN (**c**, **d**, white arrows). There is denervation oedema and atrophy of the extensor muscles (**d**, **e**, yellow arrows). **f**, **g** Axial PD (**f**) and PDFS (**g**) sequences with denervation oedema and atrophy of extensor muscles (white arrows) innervated by the PIN (not shown)

**Fig. 9:**
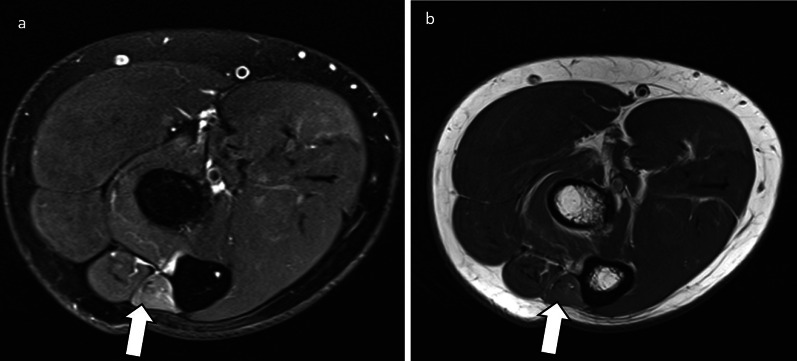
PDFS (**a**) and PD sequence (**b**) of 40-year-old male with anconeus denervation oedema (white arrows) due to radial nerve neuropathy. The anconeus is supplied by the radial nerve before it bifurcates into the deep motor and superficial sensory branches, hence the cause of neuropathy in this case will be proximal to the bifurcation

The **radial nerve** arises from the posterior cord of the brachial plexus, with roots from C5-T1. It exits the axilla via the triangular interval and travels along the radial groove of the humerus. In the arm, the radial nerve supplies motor branches to the triceps brachii, anconeus and brachioradialis and gives three sensory branches (the posterior cutaneous nerve of the arm which supplies the posterior arm, the lower lateral cutaneous nerve of the arm which supplies the lateral arm below the deltoid area, and the posterior cutaneous nerve of the forearm which supplies the posterior forearm). After entering the forearm through the cubital fossa, it divides into a deep motor branch and a superficial sensory branch. The deep motor branch descends in the posterior forearm and pierces the supinator to become the posterior interosseous nerve (PIN) which gives motor supply to the posterior forearm compartment muscles, which are primarily wrist extensors [13]. Entrapment commonly involves the deep branch and PIN. This is most common at the arcade of Frohse, a tendinous arch formed by the proximal edge of the superficial head of the supinator muscle (the course of the radial nerve through the arcade of Frohse is shown in Fig. 6). Repeated pronation and supination are suggested to induce a fibrotic process which impinges the nerve between the arcade of Frohse and the proximal radius. Less commonly, the PIN can be compressed by the deep head of the supinator muscle or the superomedial border of the extensor carpi radialis brevis [14, 15]. Clinical features of PIN entrapment include lateral elbow pain and weakness of wrist and finger extension [15]. Figures 7 and 8 illustrate different entrapment syndromes of the radial nerve. Figure 9 illustrates denervation oedema of the anconeus which indicates radial nerve pathology proximal to the bifurcation.

The **median nerve** arises from the medial and lateral cords of the brachial plexus, with roots C6-T1. It descends laterally to the brachial artery, then crosses it to sit medially halfway down the arm. It enters the cubital fossa and runs deep to the bicipital aponeurosis and anterior to the brachialis. At the elbow joint, it passes between the superficial and deep heads of the pronator teres. In the forearm, the median nerve travels between the flexor digitorum profundus and superficialis muscles, giving off two branches: the anterior interosseous nerve (AIN) which supplies the muscles in the deep anterior compartment, and the palmar cutaneous nerve which provides sensory innervation to the lateral palm. The median nerve then continues through the carpal tunnel, which is bounded superiorly by the flexor retinaculum, laterally by the trapezium and scaphoid, medially by the hook of hamate and pisiform and inferiorly by the palmar aspect of these carpal bones. Along with the median nerve, the normal carpal tunnel contains nine tendons: the flexor pollicis longus, four tendons of the flexor digitorum superficialis and four tendons of the flexor digitorum profundus. After traversing the carpal tunnel, the median nerve divides into the recurrent branch which supplies the thenar muscles, and the digital cutaneous branches which provide sensory supply to the lateral three-and-a-half digits on the palmar surface and motor supply to the lateral two lumbricals [16, 17]. The most common median nerve neuropathy is carpal tunnel syndrome, where the nerve is compressed under the flexor retinaculum. Anatomical variants, such as additional muscles in the carpal tunnel, can predispose to nerve entrapment. An example is the accessory palmaris longus. The presence of a bifid median nerve, which has a reported incidence of up to 18% and may be accompanied by a persistent median artery, has also been implicated. Mass lesions such as ganglion cysts of the wrist should also be excluded. In addition to anatomical factors, medical risk factors for carpal tunnel syndrome include diabetes, rheumatoid arthritis, hypothyroidism and obesity. However, most cases of carpal tunnel syndrome are idiopathic and typically linked with a history of repetitive or prolonged wrist flexion and extension [16]. At the distal humerus, the median nerve can be entrapped by the ligament of Struthers, which is a fibrous band connecting the congenital supracondylar spur, present in 1% of individuals, with the medial epicondyle of the humerus. Entrapment at the elbow can be due to thickening of the bicipital aponeurosis or occur between the deep and superficial heads of the pronator teres at the elbow [17].

**Fig. 10:**
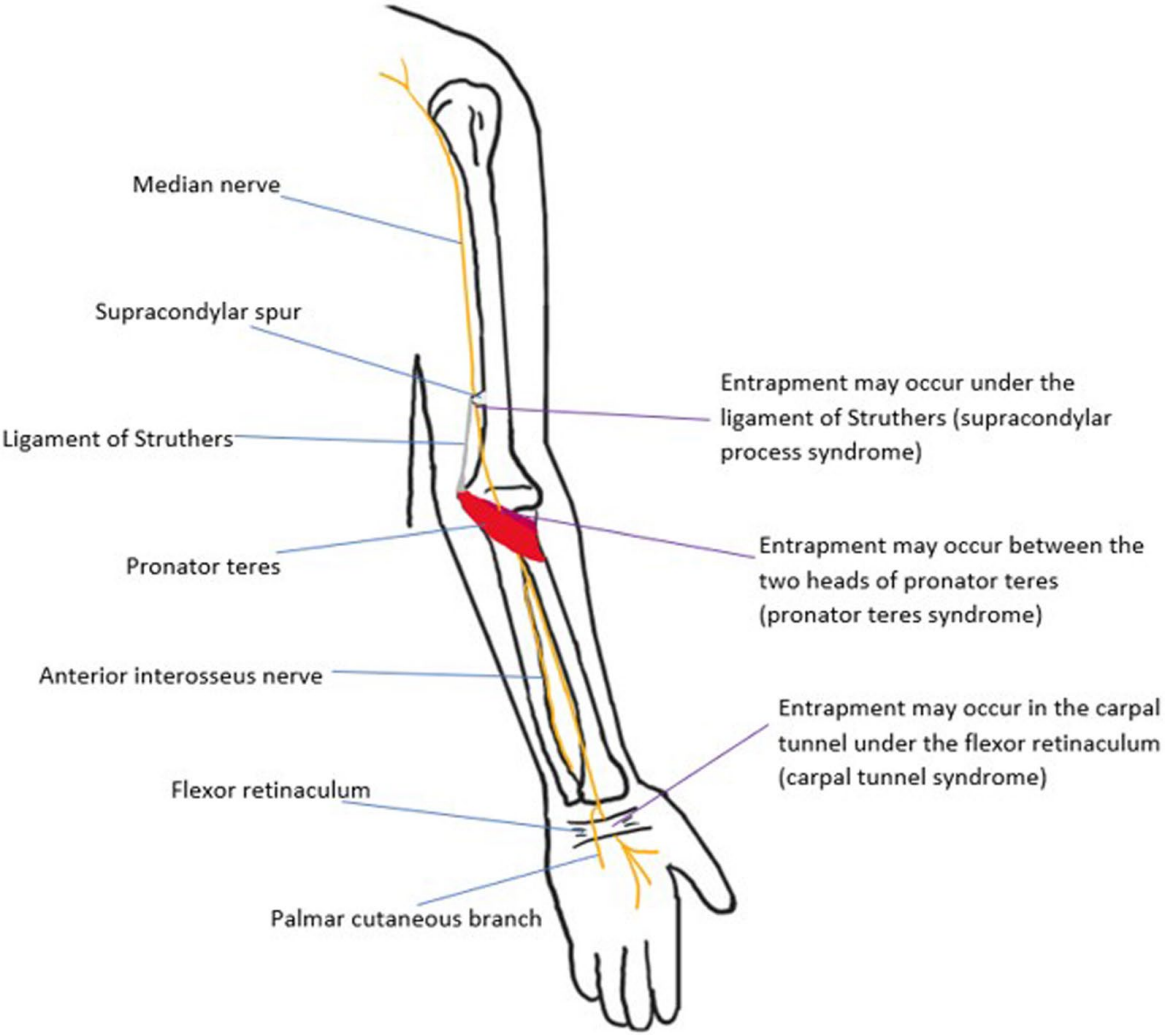
The median nerve is shown. Entrapment neuropathies of the median nerve include carpal tunnel syndrome, pronator teres syndrome and supracondylar process syndrome

**Fig. 11:**
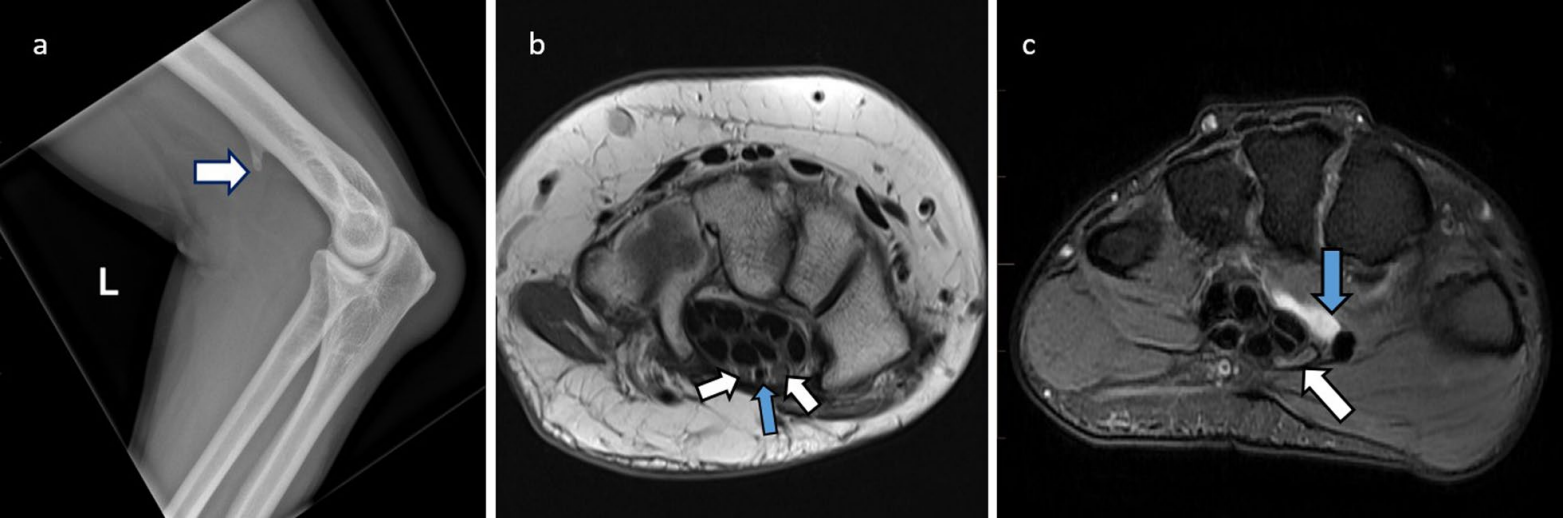
Causes of median nerve entrapment are illustrated. **a**: 56-year-old male with supracondylar spur seen on lateral elbow X-ray (white arrow). This is a known risk factor for median nerve impingement at the ligament of Struthers. **b**: Axial PD of 50-year-old female with carpal tunnel syndrome proven on electrophysiology. There is a congenital variant of bifid median nerve (white arrows) with persistent median artery (blue arrow) which can predispose to carpal tunnel syndrome. **c**: 30-year-old female with carpal tunnel syndrome. This patient has a ganglion (blue arrow) that is encroaching on the carpal tunnel. There is indentation of the median nerve with a width/height ratio of more than 3 (white arrow)

**Fig. 12:**
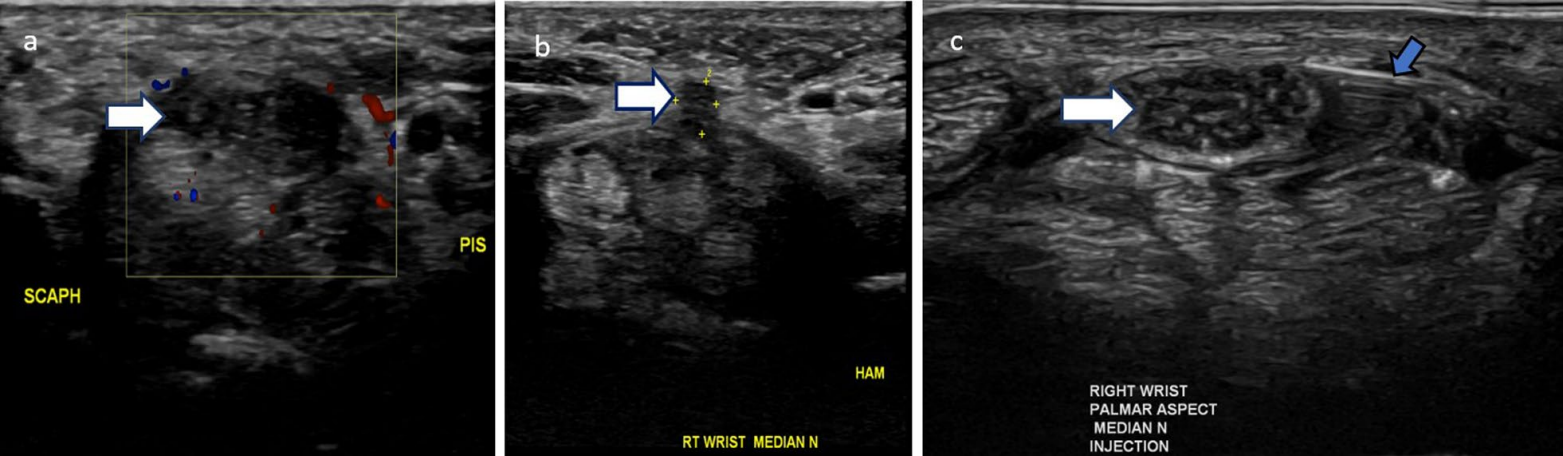
Features and management of carpal tunnel syndrome on ultrasound are illustrated. **a**, **b**: 35-year-old female with carpal tunnel syndrome. The typical ultrasound appearance for neuropathy includes hypoechoic enlargement and nerve swelling (**a**, white arrow). There is an incidental variant of the recurrent branch of the median nerve that pierces the transverse carpal ligament (**b**, white arrow). **c**: Perineural cortisone injection of the median nerve under ultrasound guidance is shown. The needle (blue arrow) is positioned superficial and to the right side of the nerve (white arrow), with injectate encircling the nerve

**Fig. 13:**
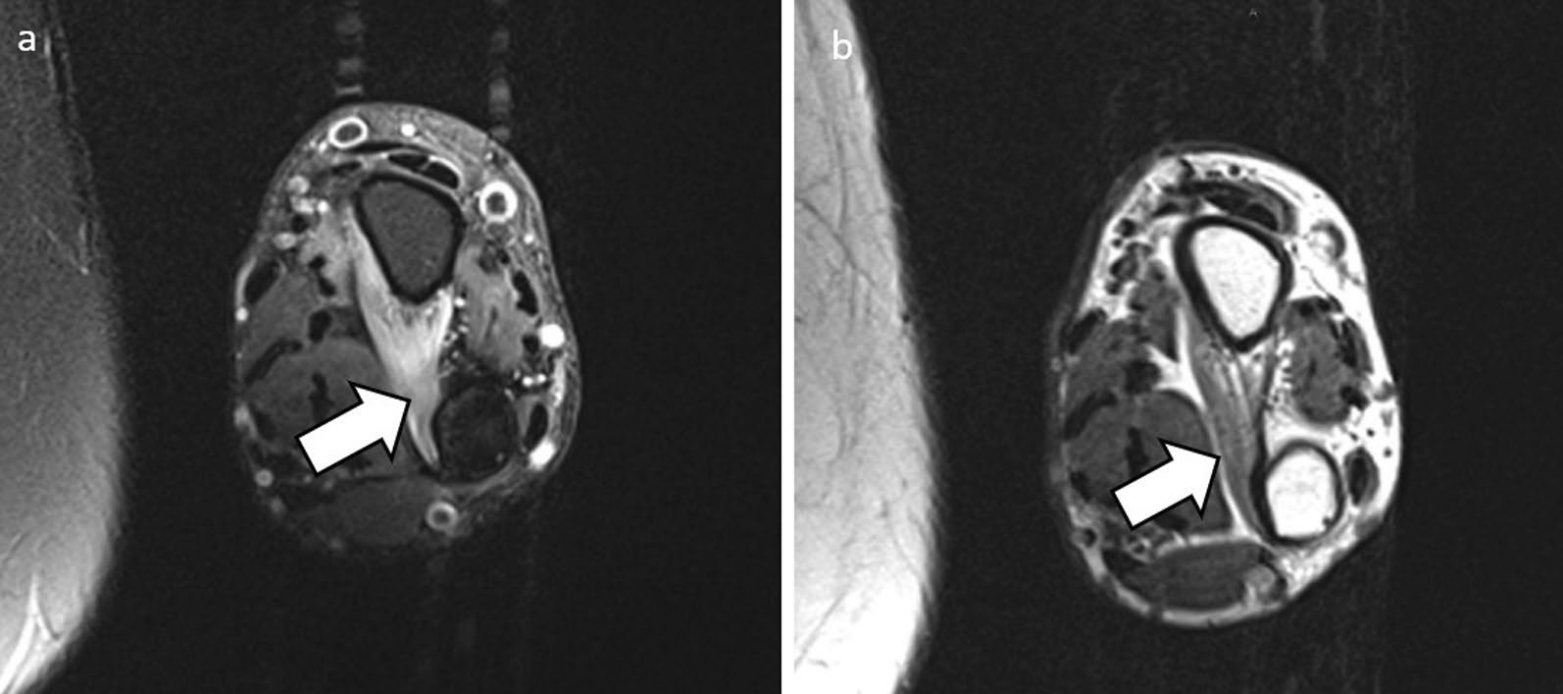
50-year-old male with flexor pollicis longus palsy. Axial PDFS (**a**) and PD (**b**) at the level of the distal forearm demonstrate atrophy and denervation oedema of the pronator quadratus (white arrows), supportive of anterior interosseous nerve (AIN) denervation. A discrete lesion or compressive cause has not been identified

The AIN branch can become entrapped by the deep head of pronator teres, the fibrous arch of flexor digitorum superficialis and by muscular abnormalities, e.g. the Gantzer muscle, the accessory head of flexor pollicis longus [17]. Pronator quadratus oedema is considered a key feature of AIN pathology, although this can also be an idiopathic finding [16]. Figure 10 shows the course of the median nerve and its entrapment neuropathies, including the carpal tunnel syndrome, pronator teres syndrome and supracondylar process syndrome. Examples of median nerve pathology are shown in Figs. 11, 12, 13. Figure 12 also illustrates ultrasound-guided cortisone injection, which is a treatment option for carpal tunnel syndrome.

**Fig. 14:**
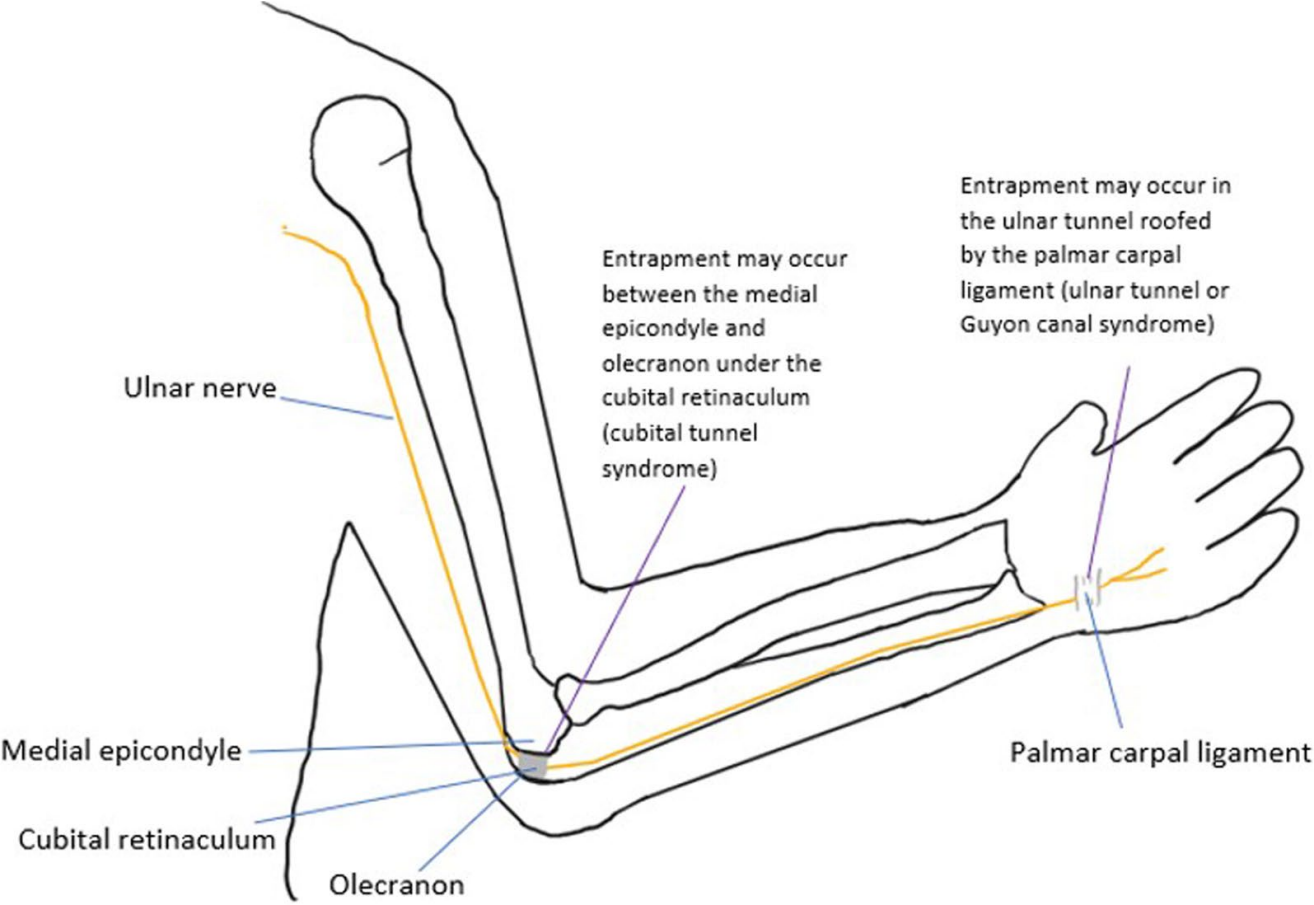
The ulnar nerve is shown. The most common entrapment neuropathies of the ulnar nerve are the cubital tunnel syndrome at the elbow, and the ulnar tunnel syndrome at the wrist

**Fig. 15:**
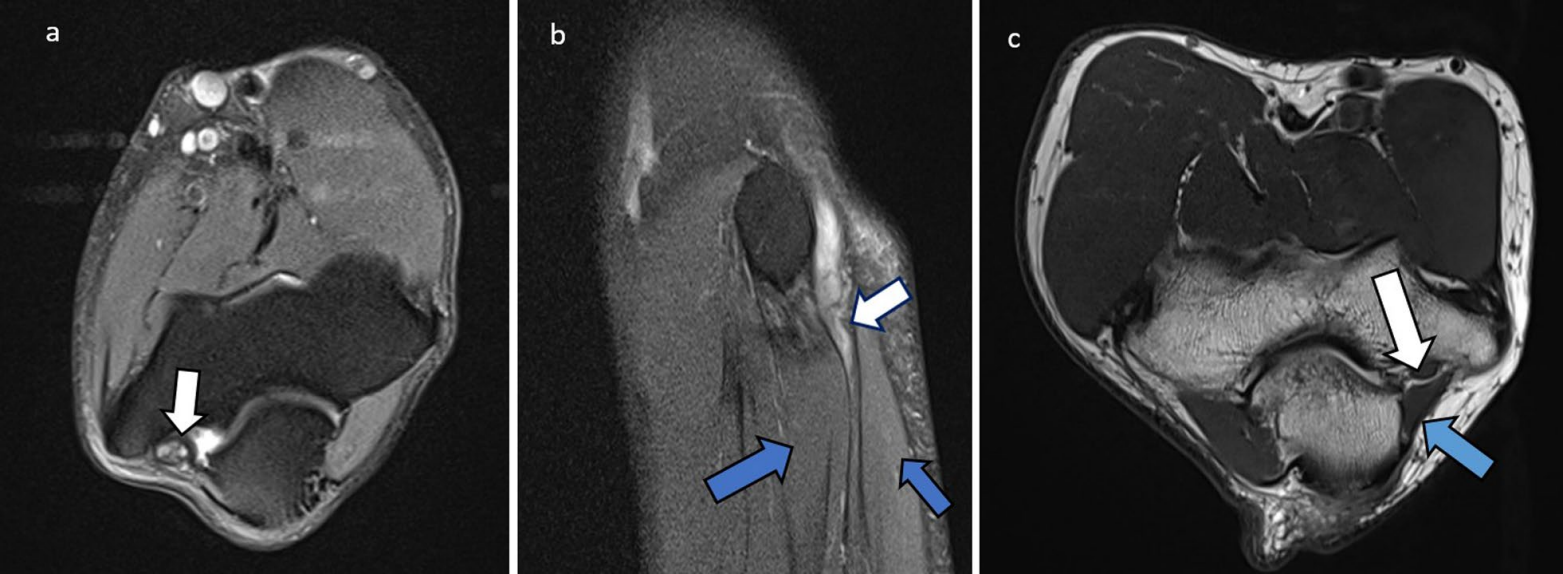
Cases of ulnar nerve entrapment at the elbow are illustrated. **a** Axial PDFS elbow in 50-year-old female with ulnar nerve neuropathy. The ulnar nerve (white arrow) returns increased signal intensity at the level of the cubital tunnel and is focally enlarged. **b** Sagittal PDFS elbow of a 54-year-old male with ulnar neuropathy. The ulnar nerve is enlarged (white arrow), with compression as it enters between the two flexor carpi ulnaris heads (blue arrows). **c** Axial PD elbow of a 60-year-old male showing the normal variant anconeus epitrochlearis (blue arrow) superficial to the ulnar nerve (white arrow) at the cubital tunnel. This can predispose to ulnar nerve entrapment

**Fig. 16:**
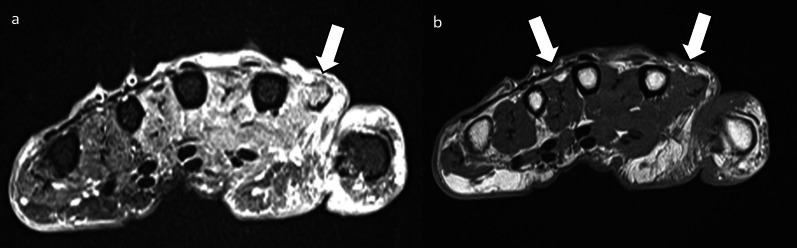
Axial PD (**a**) and PDFS (**b**) palm of an 80-year-old female. There is increased oedema and volume loss in the intrinsic muscles of the hand radially (white arrows). This is consistent with deep ulnar nerve denervation pattern

The **ulnar nerve** arises from the medial cord of the brachial plexus, with roots C8-T1. It enters the posterior compartment of the arm and passes through the cubital tunnel between the medial epicondyle and olecranon. In the forearm, it pierces the flexor carpi ulnaris, giving off a muscular branch (innervates the flexor carpi ulnaris and medial half of flexor digitorum profundus), palmar cutaneous branch (gives sensory innervation to the medial palm) and dorsal cutaneous branch (gives sensory innervation to medial hand and fingers dorsally). The ulnar nerve enters the hand via the ulnar canal (Guyon’s canal), terminating in a superficial branch (which innervates the palmar surface of the medial fingers) and deep branch (which supplies intrinsic hand muscles such as the hypothenar muscles, third and fourth lumbricals, palmar and dorsal interossei, adductor pollicis and flexor pollicis brevis). Common sites of entrapment are at the cubital tunnel and at the ulnar canal [17]. The cubital tunnel syndrome is the most common ulnar neuropathy. It is associated with the presence of the anconeus epitrochlearis, an accessory muscle present in up to 34% of the population. This muscle may compress the nerve against the medial epicondyle or olecranon of the humerus. This condition has also been linked with prolonged elbow flexion, for example in truck drivers. The ulnar canal is a less common site of entrapment, and this mainly occurs in the context of mass lesions in the canal or extrinsic compression, for example “cyclist’s palsy” which is caused by a prolonged handlebar grip [17]. Figure 14 shows the locations of ulnar canal and cubital tunnel syndrome. Figures 15 and 16 illustrate cases of ulnar nerve entrapment seen on MRI.

**Fig. 17:**
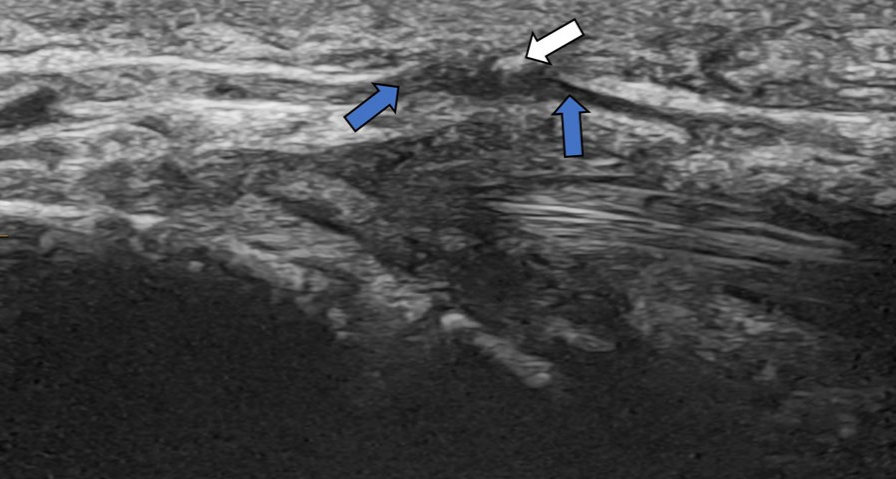
Ultrasound thumb in patient with right thumb pain post trigger finger release. There is fusiform enlargement of the digital nerve (blue arrows) palmar-radial to the site of the prior A1 pulley release, extending over a length of 5 mm. Sonographic palpation elicited the usual symptom of pain radiating down the thumb. There is an echogenic focus (white arrow) immediately superficial to the nerve which may represent suture material (or calcification). This is consistent with entrapment given the proximity to the site of prior intervention

**Digital nerves** arise from the radial, median and ulnar nerves. They are divided into palmar and dorsal branches and provide sensory and motor supply to the distal fingers. Injury to the digital nerves impairs hand dexterity and causes numbness and/or pain which radiates down the fingers. They are most commonly damaged by trauma (including surgical as shown in Fig. 17), but other causes of entrapment include inflammation, vascular malformations and neoplasms [18].

**Fig. 18:**
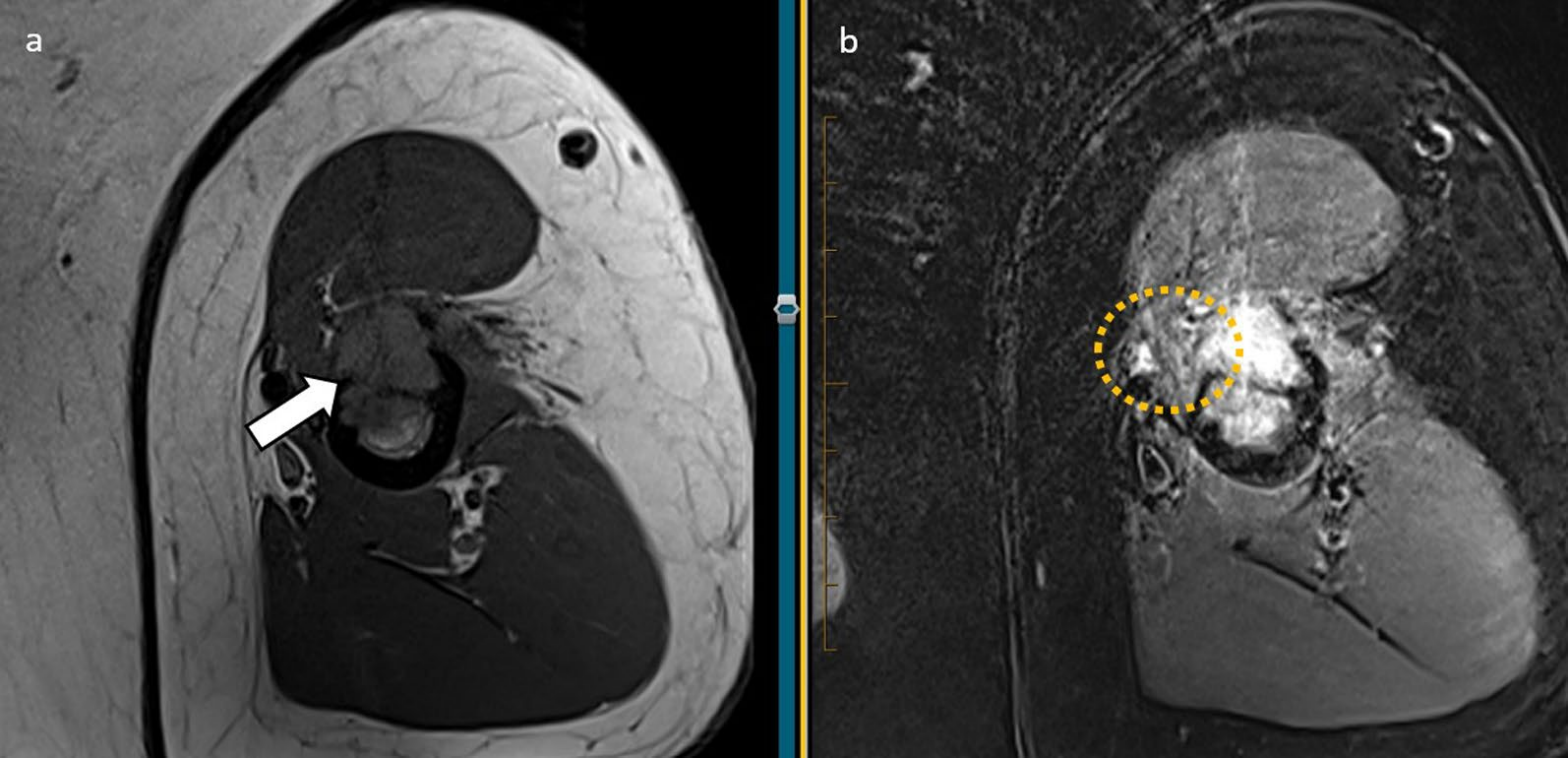
Axial T1 and PDFS of a 70-year-old male with non-small cell lung cancer. There is a lytic destructive lesion in the mid-humeral shaft with cortical disruption and extraosseous extension (white arrow) extending into the deltoid attachment and the coracobrachialis muscle. While the musculocutaneous nerve is not confidently traced due to the motion artefact, it is expected to be in close contact with the lesion. Oedema signal within coracobrachialis muscle (yellow circle) can be either due to direct muscle involvement or denervation

The **musculocutaneous nerve** arises from the lateral cord of the brachial plexus, with roots C5-C7. It exits the axilla and pierces the coracobrachialis muscle, which it supplies, before descending the flexor compartment of the upper arm superficial to the brachialis and deep to the biceps brachii, innervating these muscles. It then pierces the deep fascia and courses laterally to the biceps tendon and brachioradialis, continuing into the forearm as the lateral cutaneous nerve of the forearm, which gives sensory supply to the anterolateral forearm [19]. The coracobrachialis muscle is a common site of entrapment (Fig. 18). The lateral cutaneous nerve of the forearm can also be entrapped between the bicipital aponeurosis and brachialis fascia [19].

## Conclusion

This article describes various upper limb nerves and illustrates common sites and causes of entrapment, as well as the associated patterns of denervation oedema.

Knowledge of this helps the diagnosis and management of patients with neuropathic pain and weakness.

## Data Availability

An abbreviated adaptation of this manuscript has been submitted for poster presentation at the Royal Australian and New Zealand College of Radiologists’ Annual Scientific Meeting 2022.

## Abbreviations

AIN: Anterior interosseous nerve
MRI: Magnetic resonance imaging
MRN: Magnetic resonance neurography
PD: Proton density
PDFS: Proton density fat saturation
PIN: Posterior interosseous nerve
